# Swallowing in Alzheimer's Disease: Preliminary Results and Future Directions

**DOI:** 10.1002/alz70858_103213

**Published:** 2025-12-25

**Authors:** Megan Stradtman, Ashley N. Price, Savannah Doster, Jordan P. Sergio, Louisa I. Thompson, Alyssa N. De Vito, Jessica Alber

**Affiliations:** ^1^ Butler Hospital Memory and Aging Program, Providence, RI, USA; ^2^ Alpert Medical School of Brown University, Providence, RI, USA; ^3^ University of Rhode Island, Kingston, RI, USA; ^4^ Brown University Medical School, Providence, RI, USA; ^5^ George & Anne Ryan Institute for Neuroscience, Kingston, RI, USA

## Abstract

**Background:**

Dysphagia is a well‐known cause of morbidity and mortality in Alzheimer's disease (AD), impacting 100% of patients by the severe stage. This prevalence stands in stark contrast to 15% among community‐dwelling older adults.

Early detection of dysphagia is important for implementing effective interventions. However, dysphagia is likely underdetected in early AD. To our knowledge, cross‐sectional and longitudinal studies regarding swallowing in people with preclinical AD are nonexistent.

Here, we present preliminary results comparing cognitively unimpaired (CU) participants and those with mild cognitive impairment (MCI) on two swallowing outcome measures.

**Method:**

Forty‐two participants ages 64‐81 completed the 3‐ounce water swallow test and the 10‐Item Eating Assessment Tool (EAT‐10) in one study visit. Ten participants were MCI (5 female and 5 male; ages 64 to 81, median = 73) and 32 were CU (26 female and 6 male, ages 66 to 81, median = 71.5).

We conducted a chi‐squared test to compare the rates of passing the 3‐ounce water swallow test, and an independent‐samples median test to compare the EAT‐10 scores between the two groups.

**Result:**

Among all participants, 90.50% passed the 3‐ounce water swallow test. Those participants who did not pass were all CU; three failed due to exhibiting throat‐clearing, and one failed to drink all the water without interruption. EAT‐10 scores ranged from 0 to 3 (median = 0). All but one participant (97.61%) had EAT‐10 scores within the normal range of 2 or less. The participant who scored 3 was MCI.

The outcome of the 3‐ounce water swallow test was not associated with group assignment: χ2(1, *N* = 41)=1.382, *p* = 0.240. Median EAT‐10 score was not significantly associated with group assignment: test statistic=1.021, *p* = 0.583.

**Conclusion:**

Our findings suggest a similar prevalence of swallowing problems among our participants compared to community‐dwelling elderly in general. No significant differences between MCI and CU groups emerged.

Future work will analyze longitudinal changes in preclinical AD and controls, and between preclinical AD and symptomatic AD, to better understand the progression of swallowing disorders over the disease course.

